# Concrete-Based and Mixed Waste Aggregates in Rendering Mortars

**DOI:** 10.3390/ma13081976

**Published:** 2020-04-23

**Authors:** Samuel Roque, Cinthia Maia Pederneiras, Catarina Brazão Farinha, Jorge de Brito, Rosário Veiga

**Affiliations:** 1 Civil Engineering Research and Innovation for Sustainability (CERIS), Instituto Superior Técnico, University of Lisbon, Av. Rovisco Pais, 1-1049-001 Lisbon, Portugal; samuelroque.aa@gmail.com (S.R.); cinthiamaia@tecnico.ulisboa.pt (C.M.P.); catarina.brazao.farinha@ist.utl.pt (C.B.F.); 2 National Laboratory for Civil Engineering, Av. do Brasil 101, 1700-066 Lisbon, Portugal; rveiga@lnec.pt

**Keywords:** eco-mortars, rendering mortars, CDW, recycle, reuse, CO_2_ reduction

## Abstract

This paper presents a study of incorporation of two types of construction and demolition waste (CDW) in rendering mortars, as aggregates at 0%, 20%, 50% and 100% (by volume). Recycled concrete aggregate (RCA) and mixed recycled aggregate (MRA) were used. The former is mainly composed of cementitious waste and the latter consists of a mixture of non-segregated wastes. The performance of the cement mortars with recycled aggregates was evaluated through an extensive experimental programme. The analysis comprised workability, mechanical strength, water absorption, shrinkage, open porosity and the evaluation of durability by permeability to water under pressure after an artificial accelerated ageing test. The results are considered positive, although as the incorporation of recycled aggregates (both MRA and RCA) increased the mechanical strength, the modulus of elasticity and bulk density decreased, which leads to the production of lighter mortars that are less susceptible to cracking. The modified mortar with 20% of MRA presented the best performance, in terms of mechanical behaviour.

## 1. Introduction

The construction sector has been considered the biggest waste producer in the European Union [1]. As a result, the Waste Framework Directive 2008/98/EC of the European Parliament established that at least 70% of construction and demolition waste (CDW) should be recycled by 2020, in all the EU countries. This regulation proposes appropriate processes to collect, transport, store, treat and dispose of the waste.

Three types of recycled aggregate can be produced in recycling plants. There are: recycled concrete aggregates (RCA), mainly composed of concrete and mortar waste; recycled masonry aggregates (RMA), mainly from recycled ceramic bricks; and mixed recycled aggregates (MRA), based on a miscellany of rubble [2].

The incorporation of recycled aggregates in cementitious materials is a possible solution for the disposal of these wastes, replacing the initial constituents and, simultaneously, avoiding landfill deposition of these raw materials. The scientific community has already started researching these applications and their introduction in mortars is growing [2,3,4,5,6,7,8,9,10,11,12].

According to the European Standards EN 998-1 [13] and EN 998-2 [14], there are two main categories to classify non-structural mortars: masonry and rendering/plastering mortars. The former is used to lay and bind ceramic bricks or concrete blocks. The latter consists of coating mortars, which are used to cover external and internal walls and ceilings. The characteristics of the mortars depend on their uses. Masonry mortars contribute to the mechanical strength of the walls and rendering mortars are sacrificial layers that are applied in order to protect the substrate and provide aesthetical appearance.

In the last few years, various studies focused on the incorporation of recycled aggregates in mortars have been performed. Masonry and rendering mortars have been produced with the incorporation of three types of recycled aggregates: recycled concrete aggregate (RCA), recycled masonry aggregates (RMA), and mixed recycled aggregates (MRA).

The incorporation of RCA in masonry mortars has been developed by some authors [2,3,5,6,7,8,9,10]. The use of RCA as a replacement of the natural aggregate in rendering mortars was studied in a previous study by Neno et al. [15] and the incorporation of very fine concrete aggregates was investigated by Braga et al. [16,17]. However, in this previous research, the concrete waste used was a concrete developed in a laboratory and crushed before application on the new mortars as concrete waste aggregates. The incorporation of RCA from recycling plants (exclusively composed of concrete) into rendering mortars has not been studied in literature.

For the production of masonry mortar, recycled ceramic aggregates have been evaluated [3,4,6,11,12]. For rendering mortars, the incorporation of ceramic waste as aggregate was developed by Silva et al. [18] and Lucas et al. [19]. Furthermore, the incorporation of very fine recycled ceramics in rendering was also analysed by other authors [20,21,22].

The study of the incorporation of MRA is often neglected, due to the presence of possible contaminants, high heterogeneity, low mechanical strength compared to a natural aggregate and high water absorption ability [18,19]. This type of waste is the most common in recycling plants, and the lack of knowledge surrounding its composition makes it difficult to use. The industry of CDW management is mainly composed of small and medium-size companies, which are not well aware of the conventional procedures to maximize the production and quality of recycled aggregates [23]. MRA is strongly influenced by the processing and treatment received. Notwithstanding the lack of studies on the incorporation of MRA, some authors investigated those recycled aggregates in masonry mortar [3,4,6,11,12,24,25,26]. Only a small amount of research has evaluated the incorporation of MRA in rendering mortars. Ferreira et al. [27] analysed the role of powder content of the MRA in coating mortars produced with cement and limestone with a volumetric ratio of 1:1:6. Poon and Kou [28] also studied the use of MRA in cementitious rendering mortars. However, the composition of MRA used was based on 80% of concrete waste. MRA from CDW is very variable and heterogeneous, since it contains different quantities of each waste. MRA is influenced by the origin of the waste by the treatment process used [24]. The incorporation of poor MRA aggregated with a low volume of concrete has not been evaluated so far in literature, which is a gap in this field. Literature in this field is focused on aggregates mainly composed of concrete, even with the knowledge that the major part of construction and demolition waste are other materials such as ceramics and timber. Thus, most of the volume of construction of demolition waste still needs to be investigated in rendering mortars. In order to contribute to the knowledge about MRA, in this study, the aggregates are low in concrete (less than 50% by volume).

Taking into account the lack of studies with MRA and RCA from actual plants in rendering mortars, the main objective of this research is to analyse the use of those two types of recycled aggregates from CDW as a replacement of natural aggregate. Thus, this research is innovative and contributes not only to the knowledge about rendering mortars but also to the solution for the management of types of CDW that are available in large quantities. RCA and MRA were incorporated in ratios of 0%, 20%, 50% and 100%. In order to evaluate in depth these cementitious mortars, several tests were performed. A large number of characteristics that are very relevant for rendering mortars were analysed in this research, such as workability, flexural and compressive strength, adhesive strength, dynamic modulus of elasticity, water absorption behaviour, water vapour permeability, durability, and shrinkage.

## 2. Literature Review

From literature, it was found that the increase of the incorporation of recycled wastes as aggregate linearly decreases the workability of the mortar. Modified mortars require a greater amount of water to achieve adequate consistency. This is due to a higher water absorption and angularity of the recycled aggregates.

According to literature, the fresh and hardened bulk density of the mortars containing recycled aggregates show a decreasing trend. This is expected, bearing in mind that the particle density of recycled aggregates is lower than the natural sand’s density, due to a more porous microstructure.

Concerning the compressive strength of the modified mortars, contrasting results were obtained. In most of the studies, an increasing content of recycled aggregates results in a decrease of the mechanical strength of the mortar [5,27,28,29,30,31]. This could be explained by the fact that the compressive behaviour of the composite is highly related to the aggregate’s mechanical strength rather than to the cement matrix’s bond in the interfacial transition zone [32]. In general, the recycled aggregates [33,34] have poorer mechanical performance compared to natural particles, due to the adhered mortar and porosity of the particles.

Regarding flexural strength, there is also no consensus in the previous work. Normally, the incorporation of very fine recycled aggregates in rendering mortars presented an increase of the flexural strength, regardless of the type of waste [16,17,20,35,36]. This could be explained by the filler effect of the very fine particles. Also, the incorporation of RCA as aggregate in coating mortars presented improvements in the flexural strength [15]. On the other hand, for the incorporation of recycled aggregates for masonry mortars, slight decreases are observed in the flexural strength [2,11,12].

The flexural performance is highly influenced by the interfacial transition zone between the cement paste and the aggregates. Therefore, as recycled aggregates particles usually present a greater surface area than the natural aggregate, and they have an irregular shape and a more porous surface, these features may lead to a stronger bond to the cement matrices [15]. Another factor that may influence the flexural strength is chemical reactions, which may result from the unhydrated cement particles in RCA or even pozzolanic reactions from RMA [16,37,38].

In general, the use of recycled aggregates as aggregate in mortars decreases the modulus of elasticity [2,19,27,28]. This can be related to the amount of old mortar adhered to the recycled aggregates particles, which could lead to a greater deformability of the mortar [33,34]. This effect is considered an advantage in comparison with natural aggregate mortars, because a coating should be able to absorb the deformations imposed.

The water absorption is affected by the type of recycled aggregates incorporated. From the studies of Neno et al. [15], the incorporation of RCA slightly increased the capillary coefficient of the modified mortar, but the change was not considered significant. However, in other works, the incorporation of MRA had a significant negative influence on water absorption of the mortars [5,27,30].

As a conclusion from previous works, mortars with the incorporation of recycled aggregates from construction and demolition waste have some advantages and drawbacks over natural aggregate mortar. The effects of the use of recycled aggregates, as replacements of natural sand, is strongly correlated to their origin and the beneficiation process, which influences several properties of masonry and mortar rendering [39]. Bearing this in mind, the main contribution of this paper is to analyse the incorporation of RCA and MRA in cementitious rendering mortars, at 0%, 20%, 50% and 100% (replacing sand by volume) of each recycled aggregate type.

## 3. Materials and Methods

The aim of this work is to analyse the feasibility of the replacement of natural aggregates with recycled ones in rendering cement mortars. Two types of construction and demolition waste were used in the experimental programme: recycled concrete aggregates (RCA) and mixed recycled aggregates (MRA). The former is composed of concrete waste, and MRA is based on various constituents such as concrete, mortar, ceramic and other contaminants. Each of the recycled aggregates was incorporated in mortars replacing sand at 0% (REF), 20% (RCA20 or MRA20), 50% (RCA50 or MRA50) and 100% (RCA100 or MRA100), by volume.

The same volumetric ratio of 1:4 (cement: aggregates) was used for all the mortars produced and they are identified as presented in Table 1.

The materials used as components were firstly briefly characterized and the subsequent experimental campaign was divided in two phases. The first phase analysed the mortars performance under several tests for all the seven mortar compositions. The second phase included complementary tests on mortars selected based on the results of the first phase, in order to analyse in depth the durability and viability of the mortars as coating solutions.

### 3.1. Materials

Portland cement, silica sand and recycled aggregates from CDW were used in the production of the mortars tested. The cement used was CEM II/B-L 32.5N from the Portuguese producer Secil (Lisbon, Portugal). The natural aggregate used was siliceous sand from the Tagus River. Two Portuguese CDW recycling plants provided the recycled aggregates: RCD—Resíduos de Construção e Demolição SA (Figueira da Foz, Portugal) (RCA) and SGR—Sociedade Gestora de Resíduos S.A. (Seixal, Portugal) (MRA). Table 2 presents the bulk density of the dry materials used. The materials were previously dried for 24 h at a temperature of 105 °C. In mortar applications, the volumetric ratio is transformed in weight ratio by the multiplication of the volume ratio by the bulk density of each material. Bulk density includes the solid and air voids weight per unit of volume.

The composition of both recycled aggregates was analysed by a visual inspection, and each fraction was weighted to estimate the proportions of the different constituents. RCA samples presented 99% of cementitious materials in their composition, MRA samples were composed of several different materials, as presented in Table 3. The alternative chosen to incorporate MRA without any treatment was due to the possibility of analysing the feasibility of using this material as an aggregate, in order to avoid the need for a time-consuming and costly segregation.

**Figure 1:**
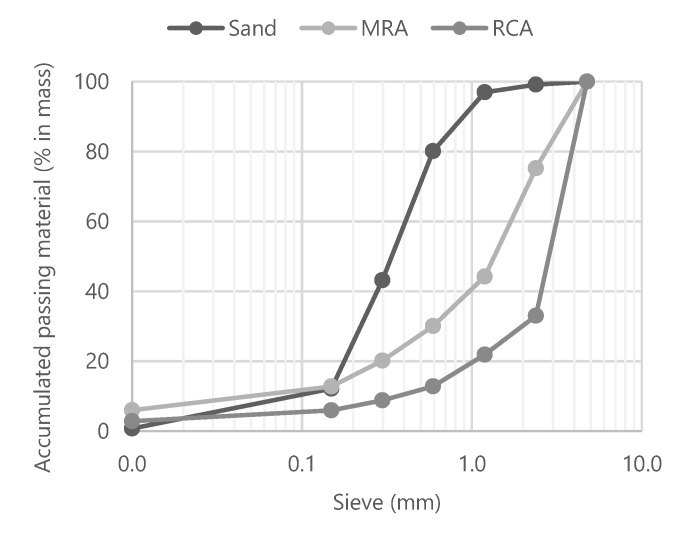
Size grading curve of the aggregates.

The size distribution of the aggregates used is presented in Figure 1. All the aggregates grain sizes were lower than 2.38 mm.

Table 4 presents the composition of the mortar mixes. From previous studies, it was found that the incorporation of other wastes (such as red ceramics [18], sanitary ware [19] or concrete waste [15]) as aggregate in rendering mortars presented an optimal incorporation ratio between 20% and 100%. Thus, in this research, the ratios of 20%, 50% and 100% of recycled aggregates were studied.

### 3.2. Methods

The tests performed on the mortars in the first and second phases of the experimental campaign are presented in Table 5.

All the specimens were submitted to a curing condition at relative humidity of 95% ± 5% and temperature of 20 ± 2 °C for two days. After the moulds were removed, the specimens were kept under the same conditions for five days. Afterwards, the relative humidity was reduced to 65% ± 5% until testing. These conditions were applied in all the tests, except for susceptibility to cracking, dimensional variation and water vapour permeability.

For the dimensional variation and susceptibility to cracking tests, the specimens were submitted to a relative humidity of 50% ± 5% and a temperature of 23 ± 2 °C, which are severe conditions for shrinkage. The cracking susceptibility was assessed by observing the possible presence of cracks and their evolution of each mortar applied on two bricks during exposure to the referred conditions. For the water vapour permeability test, the specimens were submitted to a relative humidity of 95% ± 5% and a temperature of 23 ± 2 °C for two days. After demoulding, the samples were kept at a relative humidity of 50% ± 5%.

The dynamic modulus of elasticity by frequency of resonance consists in submitting the sample to several vibration frequencies, in order to identify their resonance frequency. The dynamic modulus of elasticity is calculated based on this value using the equation hereinafter.

$$\mathrm{MEfr}=\frac{4\times L{\;}^{2}\times F^{2}\times \rho \times 10^{-6}}{g}$$

## 4. Results and Analysis

### 4.1. First Experimental Phase

#### 4.1.1. Consistency of Fresh Mortar

The consistency of the mortars in the fresh state was fixed at 160 ± 3 mm, to ensure adequate workability. From the results presented in Table 4, it was found that the type of recycled aggregate influenced the amount of water used in each mortar. RCA mortars required higher amount of water than MRA mortars. This could be explained by the presence of non-hydrated cement in the RCA waste. However, MRA mortars also demanded more water to achieve the adequate consistency compared to the reference mortar. This phenomenon is due to the composition of the MRA, which presented more porous materials than siliceous sand, such as clay and plaster.

**Figure 2:**
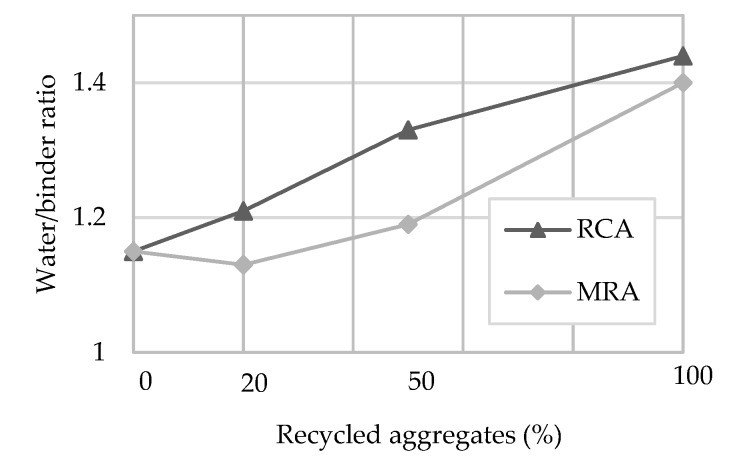
Water/binder of the produced mortars.

Figure 2 presents the water/cement ratio of each mortar produced. As the incorporation ratio of recycled aggregates increases, a greater amount of water is required to obtain the adequate plasticity, regardless of the type of recycled aggregate. The water/cement ratio was determined after the aggregates were dried, and was based on the amount of required water to achieve a proper workability to the mortar. Taking into account that renders require good plasticity for application over a vertical substrate and proper spread, the water/cement ratio obtained in this paper follows a similar trend to previous research [5,27,53].

According to Silva et al. [33], the water absorption of the recycled aggregates (RA) is higher than that of the natural aggregates, which can lead to higher absorption of the mixing water and, consequently, require a greater water content.

The incorporation of RCA in rendering mortars made by Neno et al. [15] increased the water needed to obtain the same workability as the reference mortar, although the water content required did not follow the same trend as the one found in this paper. In the research of Neno et al. [15], the incorporation of 100% of RCA required less water than the incorporation of 20% and 50% of RCA. However, compared to the reference mortar, all the modified mortars required higher content of water. This could be due to the filler effect found by the authors, which may have improved the workability of the mortars for all replacement ratios.

One of the reasons of these results is the water absorption and the shape of the recycled aggregates used [33,34]. The heterogeneity of the recycled aggregate interferes in all the properties of the mortar produced.

Several studies noticed that increasing the RA content leads to a linear increase of the water needs as occurred in this research [2,5,6,24,53].

#### 4.1.2. Bulk Density in Fresh and Hardened State

Table 6 presents the results of the bulk density. The bulk density of fresh and hardened mortar decreased as the incorporation of RA increased. This could be expected since the bulk density of the CDW particles is lower than that of the sand, due to the recycled aggregates presenting a more porous microstructure [32,33,34].

These results followed the same trend of other authors. Neno et al. [15] incorporated RCA, and it was found that the decrease of the bulk density was linear as the incorporation ratio of waste increased.

Other authors that incorporated recycled ceramic masonry waste as aggregate in mortars also found a decrease in the bulk density of the mortars in the fresh and hardened state [4,11,12,18]. This is explained by the density of RA, which usually is lower than that of the natural aggregate.

#### 4.1.3. Dynamic Modulus of Elasticity

**Figure 3:**
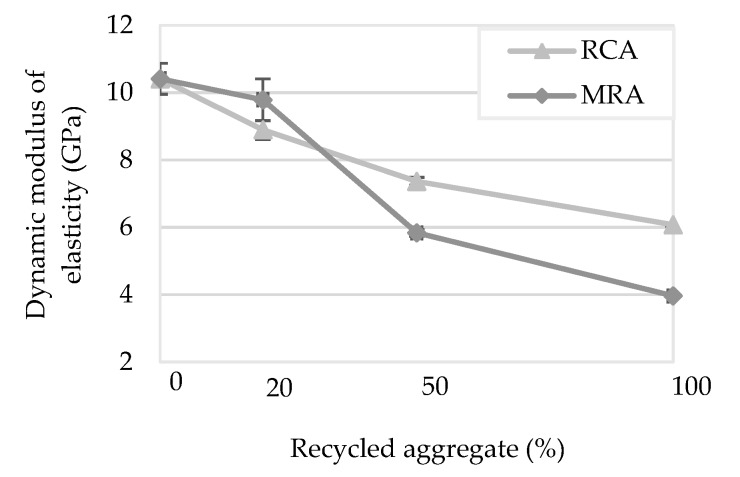
Dynamic modulus of elasticity of the mortars tested.

The dynamic modulus of elasticity was performed through the measurement of the frequency of the resonance of the samples. The results of the dynamic modulus of elasticity are presented in Figure 3. All the modified mortars obtained a lower modulus of elasticity than the control mortar. Regardless of the type of recycled aggregate, as the incorporation ratio increases, the modulus of elasticity decreases.

Mortars with 100% incorporation showed a reduction of the modulus of elasticity of approximately 62% and 42% MRA and RCA respectively compared to the reference mortar. This reduction may be favourable to the rendering mortars, since the modulus of elasticity is related to the cracking susceptibility of the mortars, since higher deformability allows lower internal stresses of the modified mortars [15,32].

The great amount of the old mortar which adhered to the particles of RCA could be the reason for the greater deformability, which led to the reduction of the dynamic modulus of elasticity. The deformation capacity of the material can be related to the dynamic modulus of elasticity, which can thus be considered an indicator of the mortar’s ability to withstand stress without cracking. MRA mortars presented a lower modulus of elasticity compared to RCA mortars. This could because this property depends directly on the density of the mortars.

The same trend was found by Ferreira et al. [27] and Poon and Kou [29], i.e., the dynamic modulus of elasticity linearly decreased as the incorporation of MRA increased. The authors justified this effect with the lower compressive strength of the recycled aggregates mortars when compared to the mortar with natural aggregates only.

#### 4.1.4. Ultrasound Pulse Velocity

**Figure 4:**
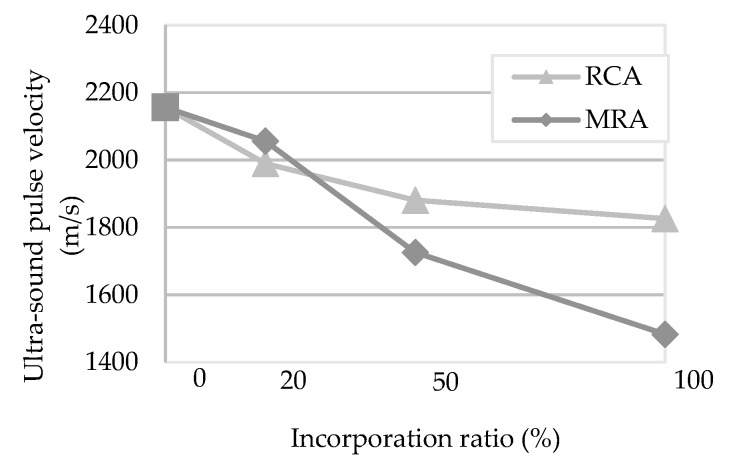
Ultrasound pulse velocity of the mortars tested by direct method at 90 days.

Figure 4 presents the results of the ultrasound pulse velocity test. This test is an indicator of the compactness of the specimens, measured by the wave propagation time between extremities (direct method) or between two points on the same surface (indirect method). A lower propagation velocity could indicate a greater volume of intercepted voids, i.e., less compactness.

For both measuring methods (direct and indirect), the incorporation of recycled aggregates decreased the pulse velocity of the mortars. These results follow the same trend of the dynamic modulus of elasticity test. They may indicate that the MRA mortars have a higher volume of pores.

#### 4.1.5. Flexural and Compressive Strength

**Figure 5:**
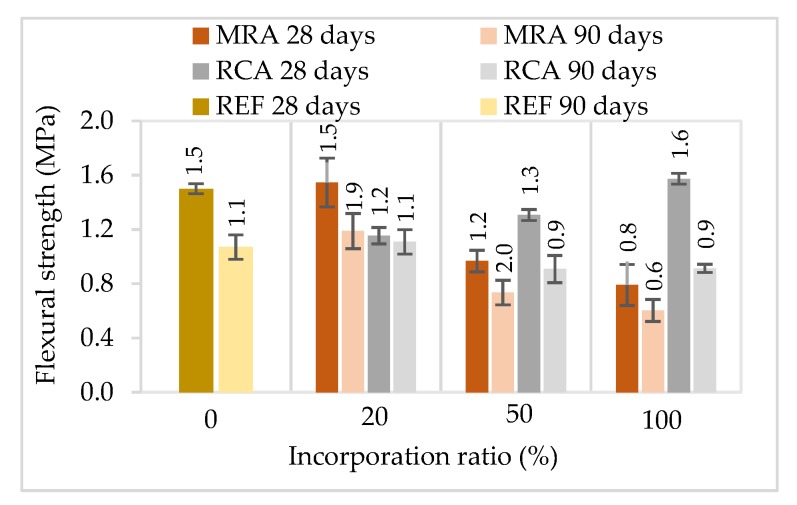
Flexural strength of the mortars tested at 28 and 90 days.

**Figure 6:**
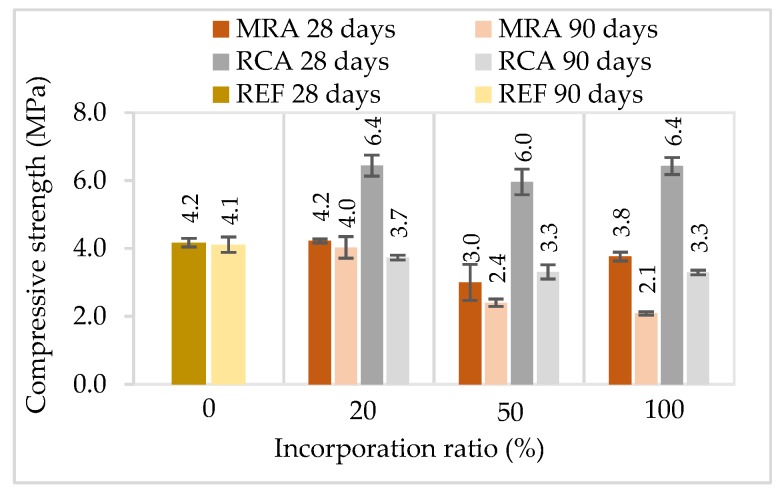
Compressive strength of the mortars tested at 28 and 90 days.

Flexural and compressive strength tests were performed at two ages, 28 and 90 days. The results are presented in Figure 5 and Figure 6. In general, the incorporation of recycled aggregates reduced mechanical strength, with the exception of RCA mortars at 28 days. Regardless of the type of recycled aggregate, mortars with 20% incorporation presented similar results compared to the reference mortar. Mortars with 50% and 100% incorporation of MRA presented a greater decrease compared to the RCA mortars.

From the results obtained, it was noticed that the standard deviation of the modified mortars is generally higher than that of the reference mortar. This is probably due to the heterogeneity of the recycled aggregate waste used. Since MRA presented in the composition several different materials, this could result in variations in the properties of the modified mortars.

The incorporation of 100% of MRA led to a decrease of the flexural and compressive strengths up to 44% and 49%, respectively, relative to the reference mortar at 90 days. The loss of compressive strength can be related mainly to the poorer mechanical properties of MRA compared those of NA [29].

Other studies that evaluated the incorporation of MRA in rendering mortars as aggregate followed the same trend as our research [24,27,28], i.e., as the replacement level increased the mechanical strength of the mortars decreased.

The incorporation of RCA led to an increase of 7% and 52% of the flexural and compressive strengths, respectively, relative to the control mortar, at 28 days. However, at the last ages tested, RCA incorporation also led to a decrease of the mechanical strength. This decrease of mechanical strengths over time can be explained by internal micro cracking in the modified mortars.

The incorporation of RCA led to better results than the use of MRA. This could be due to the concrete particles, which may enhance the bond between the cement paste and the aggregates. RCA present a more irregular and porous surface, a higher specific surface and have sharper edges, leading to a greater bond to the cement matrix [15]. Besides this improved bond, RCA may contain non-hydrated cement particles, which can undertake hydration when in contact with the mixing water [32].

Neno et al. [15] reported similar results when incorporating RCA in rendering mortars. The mortars produced with 100% of RCA increased about 89% and 58% for compressive and flexural strengths respectively relative to the reference mortar at 28 days. The authors justified those results with the lower water content in this modified mortar, which led to a lower void volume, higher cohesion and greater strength.

Other studies that incorporated very fine recycled aggregates found a significant increase in the mechanical strength of the rendering mortars, regardless of the RA used [16,17,21,35,36]. Those results are explained by the filler effect of the fine particles incorporated, and also a potential pozzolanic reactions of the CDW.

#### 4.1.6. Water Absorption by Capillarity

**Figure 7:**
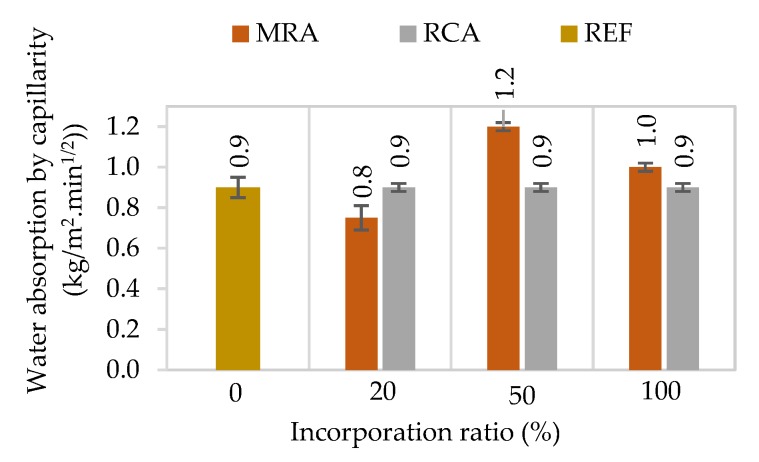
Capillarity coefficient of the mortars.

**Figure 8:**
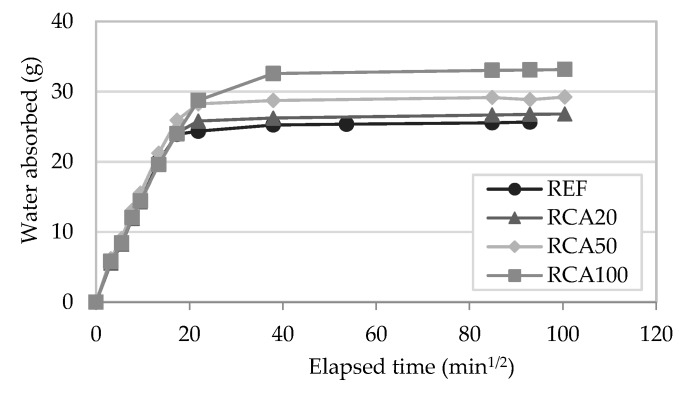
Water absorption by capillarity action tests results of recycled RCA mortars.

**Figure 9:**
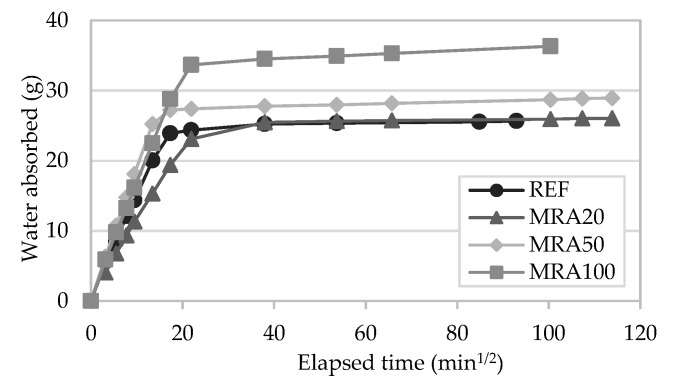
Water absorption by capillarity action tests results of MRA mortars.

Figure 7 presents the capillarity coefficient of the mortars and Figure 8 and Figure 9 present the results of the water absorption by capillarity test at 28 days. The capacity to absorb water is highly related to the durability of the renders [19]. Additionally, the coating must be able to protect the substrate from the water absorption.

The capillarity coefficient was determined according to EN 1015-18 [46], based on the water absorbed between 10 and 90 min, per unit area and square root of time. RCA mortars presented a similar behaviour in absorbing water in the first minutes of the test to that of the reference mortar, regardless of the incorporation ratio of recycled aggregate. The incorporation of MRA, in general, presented an increment of this coefficient, which is in accordance with the open porosity test results.

The incorporation of RCA presented similar results at the beginning of the test, regardless of the replacement ratio, following the same pattern as the control mortar. However, in the long term, it can be seen that the total amount of water absorbed by the mortars increased with the RCA content. Neno et al. [15] and Samiei et al. [30] had similar results. In all these studies with incorporated RCA in rendering mortars, in the long term the increase of RA ratio increased the total water absorbed.

On the other hand, the incorporation of MRA showed a greater scatter of results. MRA100 absorbed the highest amount of water. The water absorption linearly increased as the replacement ratio increased. These results agree with previous works that incorporated MRA in mortars [5,27]. This is due to the higher water absorption capacity of the recycled aggregates used relative to natural ones.

#### 4.1.7. Drying

**Figure 10:**
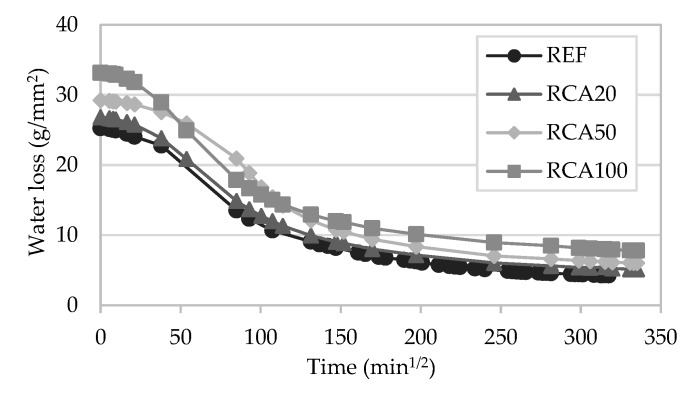
Drying curves of the RCA mortars.

**Figure 11:**
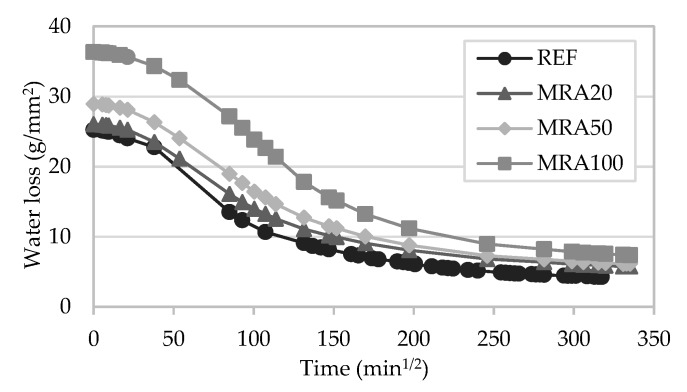
Drying curves of the MRA mortars.

The drying behaviour of the mortars could be analysed from the curves presented in Figure 10 and Figure 11. After the water absorption capillarity test, the drying time of the specimens was measured.

Mortars with incorporation of RCA presented a behaviour similar to that of the reference mortar. RCA50 showed a slower drying compared to the other mortars. From the curves, it could be observed that RCA100 presented a drying rate similar to the others, even though this modified mortar absorbed the highest amount of water of all RCA mortars.

The drying behaviour of the mortars with incorporation of MRA showed a similar pattern. MRA100 absorbed a larger volume of water than the others, but the rate of water loss was also quicker. Nonetheless, Neno et al. [15] found that the incorporation of concrete waste aggregates in mortars exhibited a similar trend to that of the reference mortar. In general, all mortars behave regularly and consistently with the results of the capillary water absorption test.

#### 4.1.8. Open Porosity

**Figure 12:**
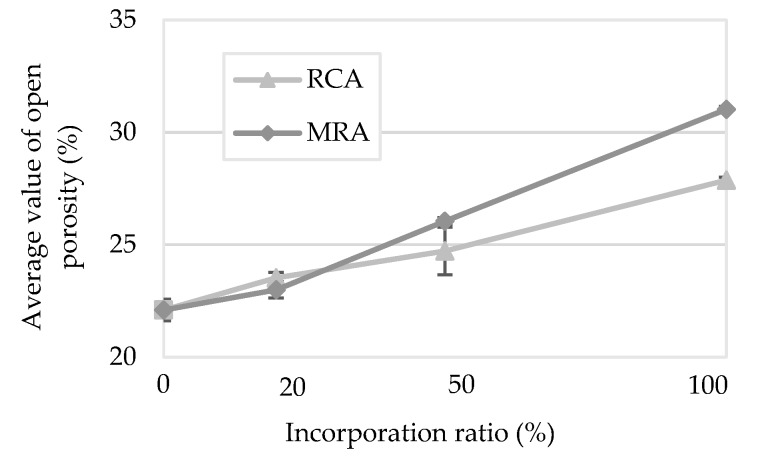
Open porosity of the mortars.

Figure 12 presents the results of the open porosity test.

The open porosity is the percentage in volume of the interconnected voids relative to the total volume of a given specimen [35]. Mortars with MRA displayed higher open porosity values than those with RCA. These results corroborate those obtained in previous tests, such as the capillary absorption tests, in which it was also the MRA mortars that presented the highest water absorption capacity.

These values are partly explained by the amount of water used in the production of mortars. In both cases (MRA and RCA), the percentage of the open porosity increased with the incorporation of RA, similar to the requirement for mixing water. However, RCA mortars required higher water content to achieve the same consistency and presented a lower open porosity than that of MRA mortars. Therefore, it is considered that other aspects, such as particle shape and composition of the RA, may influence this property as well.

Jesus et al. [17] found that the incorporation of very fine MRA increased the open porosity of the mortars. However, the modified mortars with fine RCA presented a decrease of porosity, contrasting with the results obtained in this research. This could be explained by the incorporation of very fine materials, which promotes a filler effect of the particles.

### 4.2. Second Experimental Phase

Given the previous results, one mortar of each analysed recycled aggregates was selected for a final characterization. RCA100 was chosen due to the possibility of the incorporation with a larger content of recycled aggregate, without jeopardizing the mechanical performance, enabling a total replacement of the natural aggregates. MRA20 was selected because it was the closest one to the reference mortar of the RMA mortars analysed.

#### 4.2.1. Dimensional Variation

**Figure 13:**
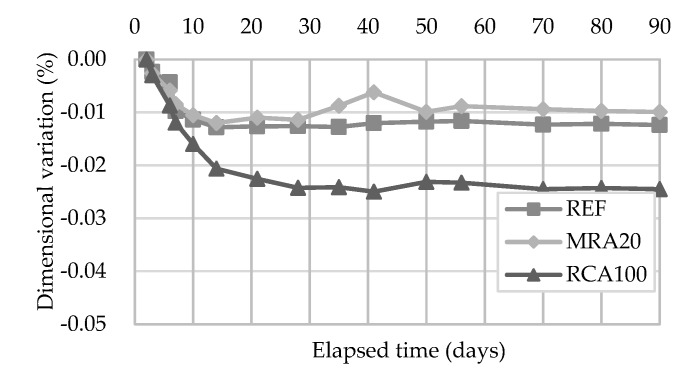
Dimensional variation of the mortars tested.

The dimensional variation (shrinkage) test intends to measure, over time, the dimensional (longitudinal) changes occurred in the mortars. The test was performed up to 90 days and the results are presented in Figure 13. The largest dimensional variation of the mortars occurred at an early age, as expected.

MRA20 presents a similar trend to that of the reference mortar. This could be due to similar porosity (found in the open porosity test) between the mortars (about 22%). The opposite occurred to RCA100, that presented an open porosity of about 28%, which is 27% higher than that of the reference mortar. Thus, RCA100 had a higher shrinkage than the control mortar.

#### 4.2.2. Water Vapour Permeability

The water vapour permeability considers the exit of the water vapour, which allows for the drying of the coating. A higher permeability to water vapour prevents problems with the interior condensation, depending on the internal structure of the material. The results of this test are presented in Table 7.

The reference mortar presented a higher water vapour resistance than the others, followed by MRA20 and RCA100. These results are consistent with those of the open porosity test, which are also compatible with the drying behaviour of the mortars. The similar trend of the results is due to the pore microstructure and communication pore channels of the mortars [2].

From the water vapour permeability test results, it was noticed that the MRA20 mortar presented a standard deviation of 2.38 ng/(m·s·Pa) which corresponded almost to 10% of the average value. The remaining mortars presented a standard deviation lower than 1%. This is attributed to the MRA waste being more heterogenous than the RCA waste, which can increase the variability of the test results obtained for those mortars.

The results obtained agree with those of Silva et al. [18], who also observed an increase in water vapour permeability with increasing ceramic waste incorporation. Lucas et al. [19] also obtained higher permeability values with the incorporation of sanitary ware as aggregate, and identified its porosity as a probable cause.

#### 4.2.3. Adherence Strength

The adherence strength test measures the interface strength between mortar and substrate, simulating a tensile stress on the coating applied on a substrate. All the mortars analysed presented a failure mechanism consisting of a combination of adhesive fracture in the interface between mortar and substrate (type A) and within the coating (type B). The results are presented in Table 8.

The reference mortar and MRA20 had similar results regarding the adherence strength. RCA100 results were 66% higher than that of the control mortar. It can be seen that the adherence strength can improve in the modified mortars. Corinaldesi et al. [2] and Kou and Poon [30] reported an increase of the bond strength between the mortar and the surface; this is due to a higher water absorption of the recycled aggregates, which can ensure a stronger interlock mechanism.

Santos et al. [54] related the adherence strength to the volume and diameter of the pores. A higher volume of the mortar’s pore diameter can increase the number of active pores of the substrate. Thus, it can improve the transport of fine particles to the porous surface, strengthening the interfacial bond.

In the study of Corinaldesi et al. [3], the incorporation of RCA and MRA in masonry mortars increased the adherence strength approximately 150% and 190%, respectively. This improvement is related to a synergic effect between concrete and ceramic waste.

Opposite results were obtained by Neno et al. [15]: when incorporating RCA in rendering mortars, the adherence strength slightly decreased. Nonetheless, the incorporation of ceramic waste in rendering mortars presented an increase in this property [18,19]. This effect could be related to the hydration of cement particles waste.

#### 4.2.4. Evaluation of Durability by the Artificial Accelerated Ageing Test

To assess the mortars behaviour over time, an artificial accelerated ageing test was carried out. This test consists of eight cycles of heat-freeze and humidity-freeze. The former consisted of submitting the specimens to 60 ± 2 °C and −15 ± 1 °C for 15 h ± 15 min. The eight humidity-freeze cycles were also performed and consisted of keeping the temperature at 20 ± 2 °C for 8 h ± 15 min with water sprinkling, and after freezing the specimens to −15 ± 1 °C for 15 h ± 15 min. After these cycles, the specimens were kept under a temperature of 20 ± 2 °C and a humidity of 65% ± 5% for two weeks.

The permeability to water under pressure and the susceptibility to cracking tests were performed after the accelerated ageing process.

##### (a) Permeability to Water Under Pressure

**Figure 14:**
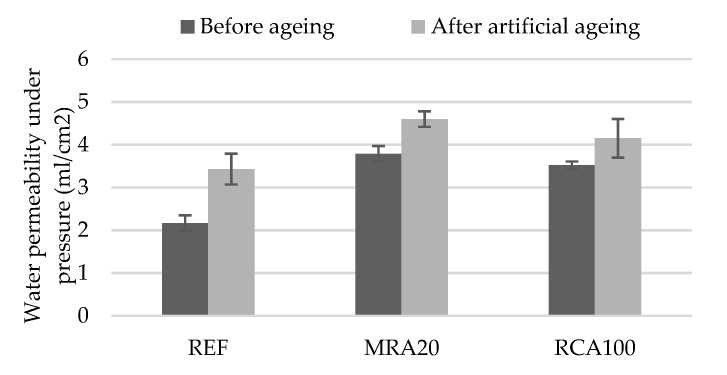
Water permeability under pressure of the mortars tested.

The permeability to water under pressure test was performed before and after the accelerated ageing, allowing evaluating the behaviour of the mortars over time. The results of the permeability to water under pressure test before ageing and after ageing are presented in Figure 14. These results corroborate the water vapour permeability, water absorption by capillarity and open porosity test results. These tests are related to the internal structure of the mortars.

From the results, it can be seen that all mortars presented a worse performance after ageing in relation to the water absorption, as expected. After the accelerated ageing procedure, the mortars absorbed a larger amount of water. Nonetheless, although mortars with RA presented higher water absorption, their loss of performance with ageing was lower than that of the REF mortar. This is attributed to the hydration of the cement particles in the RA, which once exposed to water from ageing cycles can hydrate and increase the compactness of the mortar.

Lucas et al. [19] noticed that the incorporation of sanitary ware as aggregate in rendering mortars also exhibited an increase of water permeability under pressure of the mortars. However, the increase of the water permeability under pressure of the modified mortars after the accelerated ageing process was higher that of than the reference mortar. This effect can be explained by the fact that the sanitary ware aggregates did not have dehydrated cement particles in their composition, thus the incorporation of recycled aggregates presented a higher increase in this property after ageing.

##### (b) Susceptibility to Cracking

This test consists of a visual assessment of the deterioration of the mortar surface after the accelerated ageing process, and register of any visible cracks. After the process, no visible cracks were observed in any of the mortars. The interpretation was that all the mortars had no high tendency to crack due to drying shrinkage, but the results did not allow differentiating them.

## 5. Conclusions

From the results obtained, it was concluded that the type and the volume incorporated of recycled aggregates may influence behaviour of the mortars differently. More specifically:

- The incorporation of recycled aggregates decreased the mortar’s consistency. As the replacement level increased, more water was required to achieve the same workability. A greater amount of water was required when RCA was incorporated, due to the greater surface area and the presence of non-hydrated particles;
- The fresh and hardened bulk density showed a decreasing trend as the replacement of recycled aggregate increased. This was expected due to the lower particle density of the recycled aggregates incorporated relative to the natural aggregate. In general, recycled aggregate particles presented a porous microstructure;
- Concerning the dynamic modulus of elasticity, the use of recycled aggregates improved the modified mortars to be able to withstand the deformations without fracture. MRA mortars presented a lower modulus of elasticity than that of the RCA mortars;
- Increasing the incorporation of recycled aggregates reduced the mechanical strength of the mortars, but RCA mortars showed lower decreases than MRA mortars. MRA100 obtained the greatest decrease of 44% and 49% of flexural and compressive strength, respectively;
- Modified mortars presented a higher water absorption compared to the reference mortar, showing a linear trend as the recycled aggregates content increased. Recycled aggregates are more porous than natural aggregates, which could lead to more water absorption ability of the particles;
- The water behaviour of the modified mortars was worse than that of the reference mortar. However, the type of recycled aggregate influenced the results: RCA mortars presented a better performance than MRA mortars. This is attributed to the higher water absorption capacity of the recycled aggregates, the pore microstructure and the connection of the mortars pore channels;
- The adherence strength was improved by the use of RCA. The recycled concrete particles improved the interlock in the interfacial transition zone due to the presence of non-hydrated cement;
- The modified mortars presented more heterogenous properties due to the recycled aggregates composition, which is often less uniform when compared to natural sand.

Taking into account the results obtained in this research, it can be stated that the incorporation of recycled aggregates in rendering mortars may impair the mechanical behaviour. However, the modified mortars presented a lower modulus of elasticity, which may reflect a lower susceptibility to cracking. Shrinkage and adherence resistance also presented an improvement in mortars produced with recycled aggregates. In addition, the incorporation of recycled aggregates in mortars allows a reduction in the use of non-renewable resources and also reduces deposit in landfills. Thus, the technical feasibility and sustainable contribution can be perceived from these findings.

## Figures and Tables

**Table 1:** Mortar mixes identification.

Aggregates	REF	RCA20	RCA50	RCA100	MRA20	MRA50	MRA100
Natural aggregates (NA)	100%	80%	50%	–	80%	50%	–
Recycled concrete aggregate (RCA)	–	20%	50%	100%	–	–	–
Mixed recycled aggregate (MRA)	–	–	–	–	20%	50%	100%

**Table 2:** Bulk density of the constituents used.

Constituents	Bulk Density (kg/m^3^)
Cement	1030
Natural sand	1472
RCA	1258
MRA	1162

**Table 3:** Materials identified in mixed recycled aggregate (MRA) composition.

Materials	Content (%)
Mortar and concrete	48.9
Ceramics	17.3
Rock	21.9
Glass	2.4
Tile	2.7
Plaster	4.1
Fibres	0.1
Wood	0.4
Plastic	0.1
Metal	0.4
Undifferentiated	1.8

**Table 4:** Composition of the mortar’s mixes (kg/m^3^).

Mortar	Water	Cement	Natural Sand	RCA	MRA	Water/Binder Ratio
REF	230	205.9	1177.1	0	0	1.15
RCA20	242	205.9	941.7	201.2	0	1.21
RCA50	266	205.9	588.6	502.9	0	1.33
RCA100	288	205.9	0	1005.8	0	1.44
MRA20	225	205.9	941.7	0	201.2	1.13
MRA50	238	205.9	588.6	0	502.9	1.19
MRA100	280	205.9	0	0	1005.8	1.40

**Table 5:** Composition of the mortar mixes (kg/m^3^).

Experimental Phase	Test	European Standard	Number of Samples	Specimens
**1st**	Consistency by flow table	EN 1015-3 [40]	2	Fresh mortar
	Bulk density	EN 1015-6 [41]	3	Fresh mortar
	Dry bulk density	EN 1015-10 [42]	3	Hardened mortar
	Dynamic modulus of elasticity	EN 14146 [43]	3	Hardened mortar
	Ultra-sound pulse velocity	Fe Pa 43 [44]	1	Hardened mortar
	Flexural and compressive strengths	EN 1015-11 [45]	3/6	Hardened mortar
	Water absorption by capillarity	EN 1015-18 [46]	3	Hardened mortar
	Drying	EN 16322 [47]	3	Hardened mortar
	Open porosity	NP EN 1936 [48]	3	Hardened mortar
**2nd**	Dimensional variation due to shrinkage	Cahier 2669 [49]	3	Hardened mortar
	Water vapour permeability	EN 1015-19 [50]	2	Hardened mortar
	Adherence strength	EN 1015-12 [51]	2	Brick with a layer of mortar
	Permeability to water under pressure	EN 1015-21 [52]	2	Brick with a layer of mortar
	Susceptibility to cracking	Internal protocol	2	Brick with a layer of mortar

**Table 6:** Bulk density of the mortars (kg/m^3^).

Mortar	Bulk Density of Fresh Mortars (kg/m^3^)	Dry Bulk Density of Hardened Mortars (kg/m^3^)
REF	1952 ± 4	1836 ± 10
RCA20	1910 ± 6	1810 ± 7
RCA50	1859 ± 12	1730 ± 12
RCA100	1770 ± 3	1660 ± 10
MRA20	1838 ± 24	1777 ± 10
MRA50	1765 ± 0	1650 ± 8
MRA100	1698 ± 2	1562 ± 9

**Table 7:** Water vapour permeability of the mortars tested.

Mortar	Water Vapour Permeability (ng/(m·s·Pa))	Standard Deviation (ng/(m·s·Pa))	Air Layer Thickness (m)
REF	17.95	0.18	0.21
MRA20	23.94	2.38	0.16
RCA100	25.38	0.17	0.15

**Table 8:** Adherence strength of the mortars tested.

Mortar	Adherence (MPa) (Average)	Standard Deviation (MPa)	Fracture Pattern
REF	0.54	0.11	A/B
MRA 20	0.48	0.04	A/B
RCA100	0.90	0.07	A/B

Note: Fracture pattern A—Adhesive fracture (in the interface mortar/substrate); Fracture pattern B—Cohesive fracture (within the coating); Fracture pattern C—Cohesive fracture (in the substrate).
